# Nitrogen supply modulates nitrogen remobilization and nitrogen use of wheat under supplemental irrigation in the North China Plain

**DOI:** 10.1038/s41598-020-59877-5

**Published:** 2020-02-24

**Authors:** Xuejiao Zheng, Zhenwen Yu, Yongli Zhang, Yu Shi

**Affiliations:** Key Laboratory of Crop Ecophysiology and Farming System, Ministry of Agriculture, Shandong Agricultural University, Tai-an 271018 Shandong, China

**Keywords:** Plant ecology, Plant physiology

## Abstract

Excessive nitrogen (N) input and irrigation exacerbate N leaching in winter wheat production in the North China Plain (NCP). To explore the optimal N for better N remobilization and higher N utilization of wheat under water-saving irrigation will be conductive to less environmental contamination. A field experiment was conducted at 300 (N_300_), 240 (N_240_), 180 (N_180_), and 0 (N_0_) kg N ha^−1^ of N application under supplemental irrigation (SI) that brought the relative soil water content (RSWC) to 70% at jointing and 65% at anthesis. Compared with N_0_, N_180_ improved the free amino acid content in the flag leaf and grain after anthesis, dry matter and plant N accumulation at maturity, N translocation amount of vegetable organs and its contribution to grain from anthesis to maturity. Compared to N_240_ and N_300_, N_180_ increased the N translocation efficiency of vegetable organs, and reduced the soil NO_3_-N residue in the 60–180 cm soil layer, which contributing to no significant reduction in grain yield and grain protein yield, but higher grain N recovery efficiency (^G^RE_N_), N recovery efficiency (RE_N_), and N partial factor productivity (PFP_N_). Positive relationships were found between leaf N translocation efficiency and grain yield, grain protein yield, PFP_N_, ^G^RE_N_, and RE_N_. Therefore, N_180_ is appropriate to obtain a steady grain yield over 7.5 t ha^−1^ for at least 2 years under SI based on RSWC in the NCP.

## Introduction

As a main grain-producing area, the output of the North China Plain (NCP) accounts for approximately 61% of China’s total wheat production^1^. However, to continuously maximize crop yield, the traditional nitrogen (N) cultivation practices of farmers are high, at up to ≥300 kg N ha^−1^, which threatens the ecological safety of the area^2–4^. A constant increase in N input decreases the N use efficiency, leading to large amounts of nitrate N leaching in soil, gaseous N losse and environmental N pollution^5–7^.

N plays an essential role in plant growth and, therefore, to agricultural production^8^. N yield is derived from two sources: (1) stored N in vegetative parts before anthesis, and (2) N uptake during grain filling^9,10^. The amount of N remobilized to the grain largely depends on N stored at anthesis, and post-anthesis N remobilization efficiency of wheat increased from 67 to 71% when N input increased from 109 to 227 kg N ha^−1^ ^11,12^. N distribution in wheat tissues is closely related to the productivity of its canopy, and high yield or high-quality grain in wheat requires the application and uptake of high levels of N fertilizer^13,14^. However, increasing N application does not always lead to a commensurate increase in grain yield^15^. Additionally, water availability affects the chemical form of N in all phases of the N cycle^16^. Previous studies have shown that conditions of moderate soil moisture are beneficial for N availability and uptake, and hence plant growth and yield^17–19^. 100% full irrigation increased N accumulation in grain at maturity by 15.9% and grain yield by 17.1%, respectively, compared with 50% full irrigation^20^. The use of an appropriate irrigation regime can enhance N uptake and reduce N loss^21^. Thus, N supply combined with optimization of supplemental irrigation (SI) is necessary to increase N uptake and reduce soil NO_3_-N leaching.

Previous studies have applied water-saving technology to determine the amount of SI based on the relative soil water content (RSWC), considering changes in precipitation and soil water availability, which is an important way to achieve high yield and water use efficiency in winter wheat production^22,23^. However, few studies have focused on the response of N uptake, translocation, and N use following N fertilizer application under conditions of SI based on the RSWC. Therefore, in this study, we applied N at four rates with a target RSWC at jointing and at anthesis. The objectives of the present study were: (1) to clarify the changes in N accumulation and translocation in individual organs of wheat in response to N input; (2) to quantify the dynamics of soil NO_3_-N content in the 0–200 cm soil layer, and (3) to determine the optimal rate of N application under SI based on RSWC to maintain higher grain yield and higher N utilization in the NCP.

## Results

### Free amino acid content in flag leaves

**Figure 1:**
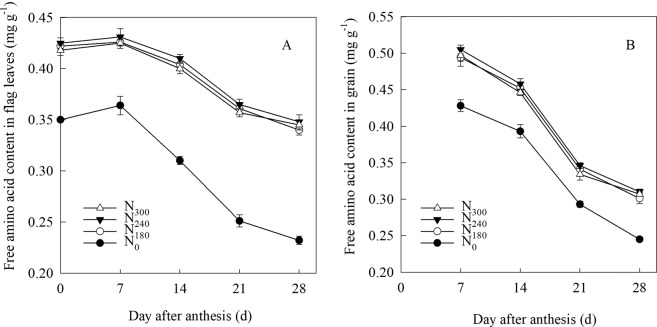
Changes of free amino acid content in wheat flag leaf (**A**) and grain (**B**) under different N treatments in 2016/2017. N_300_, N_240_, N_180_ and N_0_ represent N application rate at 300, 240, 180 and 0 kg N ha^−1^, respectively. Vertical bars represent standard deviation of the means.

N input significantly influenced the free amino acid content in flag leaves after anthesis (Fig. 1A) in 2016/2017. Compared with N_0_, N_180_ resulted in a higher content of free amino acids in flag leaves at 0, 7, 14, 21, and 28 days after anthesis, and there was no significant differences in those between N_180_, N_240_, and N_300_. N_0_ significantly decreased the free amino acid content in grain at 7, 14, 21, and 28 days after anthesis compared with other N inputs (Fig. 1B). No significant differences in free amino acid content were observed in grain after anthesis between the N_180_, N_240,_ and N_300_ treatments.

### Dry matter and plant N accumulation

Dry matter accumulation (DMA) of wheat at different stages was significantly affected by N and year during the wheat growing seasons studied; however, N × year interaction had no significant effects on DMA at anthesis and maturity (Table 1). The DMA in N_180_ significantly increased by 25.23, 21.88, 24.94, 12.35, and 21.74%, respectively, compared with N_0_ in the pre-winter, revival and jointing, anthesis, and maturity stages in 2015/2016. When N input increased to N_240_ and N_300_, there were no significant increases in DMA. Higher DMA in the pre-winter, revival and jointing, anthesis, and maturity stages in 2016/2017 was obtained with N_180_ compared with N_0_; there were no significant differences in this variable between N_180_, N_240_, and N_300_.

**Table 1:** Dry matter accumulation and plant N accumulation of wheat at different growth stages under different N treatments in 2015/2016 and 2016/2017 (kg ha^−1^). N_300_, N_240_, N_180_ and N_0_ represent N application rate at 300, 240, 180 and 0 kg N ha^−1^, respectively. Different letters indicate statistical significance at P < 0.05 among treatments. *Significant at P < 0.05. **Significant at P < 0.01. ***Significant at P < 0.001. ns, not significant, P ≥ 0.05.

Year	Treatment	Dry matter accumulation (t ha^−1^)					Plant N accumulation (kg ha^−1^)				
		Pre-winter	Revival	Jointing	Anthesis	Maturity	Pre-winter	Revival	Jointing	Anthesis	Maturity
2015/2016	N_300_	0.870a	1.99a	4.93a	11.5a	17.1a	30.7a	78.9a	156a	215a	262ab
	N_240_	0.862a	1.98a	5.00a	11.6a	17.3a	30.3ab	79.4a	158a	219a	272a
	N_180_	0.834a	1.95a	4.86a	11.1a	16.8a	29.0b	75.4a	154a	208a	256b
	N_0_	0.666b	1.60b	3.89b	9.88b	13.8b	21.2c	56.5b	125b	135b	194c
2016/2017	N_300_	0.729a	2.52a	5.65a	12.2b	17.3b	30.7a	94.9a	177a	214b	279b
	N_240_	0.719a	2.52a	5.77a	12.8a	18.2a	30.2a	94.2a	183a	224a	297a
	N_180_	0.689a	2.47a	5.67a	12.3ab	17.7ab	28.4a	90.7a	181a	213b	285ab
	N_0_	0.603b	1.82b	4.24b	10.8c	14.9c	24.6b	64.5b	136b	146c	206c
ANOVA											
Nitrogen (N)		***	***	***	***	***	***	***	***	***	***
Year (Y)		***	***	***	***	***	ns	***	***	*	***
N × Y		*	***	**	ns	ns	*	ns	ns	ns	ns

N significantly affected N accumulation in plants at different stages, and there were no interactions between year and N treatment (except for N accumulation in plants at pre-winter) (Table 1). In 2015/2016, N_180_ significantly increased plant N accumulation in the pre-winter, revival and jointing, anthesis, and maturity stages compared with N_0_, and no differences were observed between N_180_, N_240_, and N_300_. In 2016/2017, although N_240_ treatment resulted in the highest plant N accumulation at anthesis, the plant N accumulation in the pre-winter, revival and jointing, and maturity stages was significantly increased up to N_180_ compared with N_0,_ with no differences between N_180_, N_240_, and N_300_ treatments.

### N translocation from anthesis to maturity

The N translocation amount, N translocation efficiency, and N contribution to grain in stem and sheath (STS), and leaf from anthesis to maturity were significantly affected by N and year (except for N translocation amount in the STS) (Table 2). Compared with N_0_, N translocation amount in the STS and leaf following application of N_180_ were increased by 61.13 and 85.99% in 2015/2016, and 61.21 and 62.75% in 2016/2017, respectively, with no obvious differences between N_180_, N_240_ and N_300_. N_180_ resulted in the highest N translocation efficiency in the STS and leaf among N treatments in both years. Compared with N_0_, N_180_ significantly increased the STS and leaf N contribution to grain; there were no further increases with an increase in N application up to N_240_ and N_300_.

**Table 2:** N translocation of vegetable organs in wheat and its contribution to grain from anthesis to maturity under different N treatments in 2015/2016 and 2016/2017. N_300_, N_240_, N_180_ and N_0_ represent N application rate at 300, 240, 180 and 0 kg N ha^−1^, respectively. STS, Stem and sheath. Different letters indicate statistical significance at P < 0.05 among treatments. *Significant at P < 0.05. **Significant at P < 0.01. ***Significant at P < 0.001. ns, not significant, P ≥ 0.05.

Year	Treatment	N translocation amount (kg ha^−1^)		N translocation efficiency (%)		N contribution to grain (%)	
		STS	Leaf	STS	Leaf	STS	Leaf
2015/2016	N_300_	79.6a	57.3a	78.8b	80.4b	41.0a	29.5a
	N_240_	82.9a	58.5a	79.0b	79.8b	41.0a	28.9a
	N_180_	79.6a	58.4a	80.4a	82.3a	40.8a	29.9a
	N_0_	49.4b	31.4b	75.6c	76.4c	33.5b	21.2b
2016/2017	N_300_	77.7b	50.4a	72.2b	81.5b	35.6a	23.1a
	N_240_	84.8a	52.0a	73.0b	81.6b	36.3a	22.2a
	N_180_	82.7a	49.8a	74.7a	83.4a	36.4a	21.9a
	N_0_	51.3c	30.6b	70.3c	79.6c	31.5b	18.8b
ANOVA							
Nitrogen (N)		***	***	***	***	***	***
Year (Y)		ns	***	***	***	***	***
N × Y		ns	*	ns	ns	ns	**

### Distribution of N in different organs

**Figure 2:**
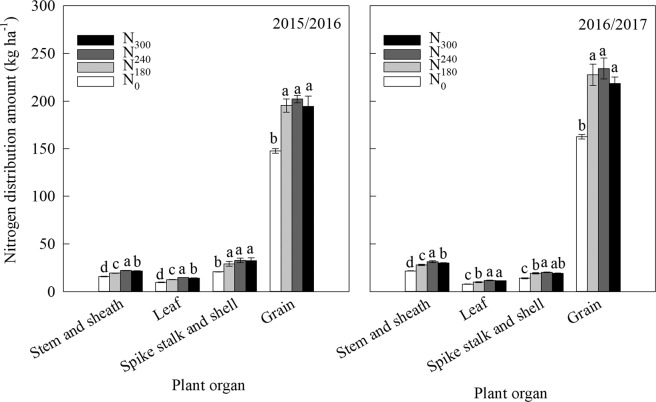
Amount of N distribution in wheat at maturity under different N treatments in 2015/2016 and 2016/2017 (kg ha^−1^). N_300_, N_240_, N_180_ and N_0_ represent N application rate at 300, 240, 180 and 0 kg N ha^−1^, respectively. Vertical bars represent standard deviation of the means. Different letters indicate statistical significance at P < 0.05 among treatments.

As shown in Fig. 2, N distribution was highest in the grain at maturity, and lowest in the leaf in both years. N_0_ resulted in the lowest amount of N distributed in different organs. The highest amount of N was distributed in the stem and sheath following treatment with N_240_, followed by N_300_, and then N_180_ in both years. N_180_ decreased the amount of N distributed in the leaf, which decreased by 15.46 and 10.51% with N_240_ and N_300_, respectively, in 2015/2016, and decreased by 15.13 and 12.82% in 2016/2017. The amount of N distributed in the spike stalk and shell, and grain with N_180_ increased by 40.03 and 32.26% in 2015/2016, respectively, and 36.97 and 39.94% in 2016/2017 compared with N_0_, with no obvious differences observed between the N_240_ and N_300_ groups.

### Soil NO_3_-N residue

**Figure 3:**
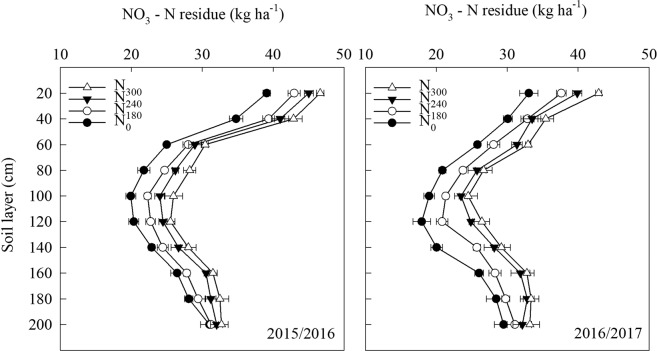
Soil NO_3_-N residue of wheat at maturity under different N treatments in 2015/2016 and 2016/2017 (kg ha^−1^). N_300_, N_240_, N_180_ and N_0_ represent N application rate at 300, 240, 180 and 0 kg N ha^−1^, respectively. Vertical bars represent standard deviation of the means.

N input significantly affected soil NO_3_-N residue in the 0–200 cm soil layer at maturity (Fig. 3). The lowest soil NO_3_-N residue in the 0–200 cm layer was obtained with N_0_ in both years. In 2015/2016, N_300_ resulted in the highest soil NO_3_-N residue in the 0–20 cm layer, followed by N_240_, and then N_180_. No obvious differences were observed among N application treatments in the 20–40 cm layer. Soil NO_3_-N residue in the 60–180 cm layer with N_180_ was lower than that with N_240_ and N_300_. In 2016/2017, the soil NO_3_-N residue in the 0–20 cm layer was similar to that observed in 2015/2016. N_180_ and N_240_ decreased the soil NO_3_-N residue in the 20–40 cm layer compared with N_300._ N_180_ significantly decreased the soil NO_3_-N residue in the 40–180 cm soil layer compared with N_240_ and N_300_, and no obvious differences were observed among N treatments in the 180–200 cm layer.

### Grain yield, grain protein yield, and N utilization

**Figure 4:**
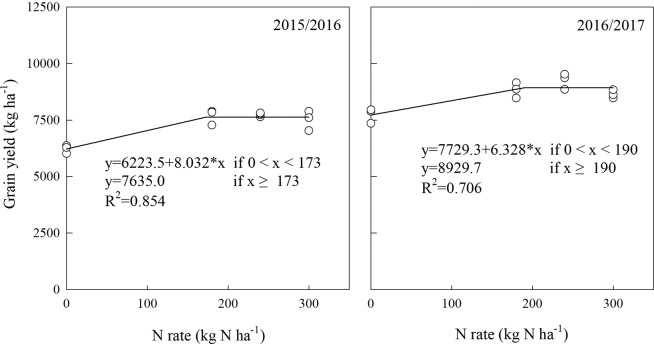
Linear-plateau model fitted for wheat yield as function of N application rate in 2015/2016 and 2016/2017.

The spike number, 1000-grain weight and grain yield were significantly affected by N rates and showed significant yearly variations, while there were no significant N × year interactions on grain number per spike and grain yield (Table 3). Compared with N_0_, N_180_ increased the spike number by 4.66 and 13.49% in 2015/2016 and 2016/2017, respectively, with no obvious differences between N_180_, N_240_ and N_300_. No substantial differences were found in grain number per spike among all N treatments, and the highest 1000-grain weight was observed with N_0_ in both years. The response of grain yield to the applied N rate fit a linear-plateau model (Fig. 4), and sharply increased up to N_180,_ by 23.31 and 14.23% over N_0_ in 2015/2016 and 2016/2017, respectively. No obvious differences were detected in grain yield among the N_180_, N_240_, and N_300_ treatments in either year.

**Table 3:** Yield components, yield, grain protein yield and N utilization of wheat under different N treatments in 2015/2016 and 2016/2017. N_300_, N_240_, N_180_ and N_0_ represent N application rate at 300, 240, 180 and 0 kg N ha^−1^, respectively. PFP_N_, N partial factor productivity, ^G^RE_N_, Grain N recovery efficiency, RE_N_, N recovery efficiency. Different letters indicate statistical significance at P < 0.05 among treatments. *Significant at P < 0.05. **Significant at P < 0.01. ***Significant at P < 0.001. ns, not significant, P ≥ 0.05.

Year	Treatment	Spike number (m^−2^)	Grain number per spike	1000-grain weight (g)	Grain yield (t ha^−1^)	Grain protein concentration (%)	Grain protein yield (t ha^−1^)	PFP_N_ (kg kg^−1^)	^G^RE_N_ (%)	RE_N_ (%)
2015/2016	N_300_	592a	33.4a	42.2b	7.51a	14.8ab	1.11a	25.0c	15.6b	22.7b
	N_240_	594a	33.4a	42.4b	7.73a	14.9a	1.15a	32.2b	22.7a	32.3a
	N_180_	584a	33.1a	43.2ab	7.67a	14.5b	1.11a	42.6a	26.5a	34.6a
	N_0_	558b	32.7a	43.7a	6.22b	13.5c	0.84b	—	—	—
2016/2017	N_300_	616a	33.4a	40.9b	8.65a	14.4a	1.24b	28.8c	18.6b	24.2b
	N_240_	637a	34.0a	41.0b	9.25a	14.4a	1.34a	38.5b	29.8a	38.0a
	N_180_	614a	33.7a	41.5b	8.83a	14.7a	1.30ab	49.1a	36.1a	43.7a
	N_0_	541b	32.9a	44.7a	7.73b	12.0b	0.93c	—	—	—
ANOVA										
Nitrogen (N)		***	ns	***	***	***	***	***	***	***
Year (Y)		**	ns	**	***	**	***	***	**	**
N × Y		**	ns	**	ns	**	ns	ns	ns	ns

The grain protein concentration and grain protein yield were significantly affected by N and year (Table 3). N_180_ resulted in a higher grain protein concentration and grain protein yield than N_0_, and no obvious difference was found between N_240_ and N_300_. The highest PFP_N_, ^G^RE_N_, and RE_N_ were obtained with N_180_. When N was increased to N_240_, the PFP_N_ with N_240_ decreased by 24.41 and 21.59% compared with N_180_, respectively, in 2015/2016 and 2016/2017, and there were no significant increases in ^G^RE_N_ and RE_N_. The lowest PFP_N_, ^G^RE_N_, and RE_N_ were found with N_300_.

### Correlation studies

Grain yield was positively correlated with stem and sheath N translocation amount (r = 0.650) and leaf N translocation efficiency (r = 0.750) (Table 4). Grain protein yield was positively correlated with stem and sheath N translocation amount (r = 0.871), leaf N translocation amount (r = 0.676), and leaf N translocation efficiency (r = 0.775), but not with stem and sheath N translocation efficiency. ^G^RE_N_ was positively correlated with stem and sheath N translocation amount (r = 0.569), leaf N translocation efficiency (r = 0.642), grain yield (r = 0.547), grain protein yield (r = 0.580), and strongly positively correlated with PFP_N_ (r = 0.877) and RE_N_ (r = 0.951).

**Table 4:** Correlation analysis of N translocation, grain yield, grain protein yield, N partial factor productivity (PFP_N_), grain N recovery efficiency (^G^RE_N_) and N recovery efficiency (RE_N_) in 2015/2016 and 2016/2017 (n = 24). STSNT, Stem and sheath N translocation amount; LNT, Leaf N translocation amount; STSNE, Stem and sheath N translocation efficiency; LNE, Leaf N translocation efficiency; GY, Grain yield; GPY, Grain protein yield. *Correlation is significant at the 0.05 level (2-tailed). **Correlation is significant at the 0.01 level (2-tailed).

	STSNT	LNT	STSNE	LNE	GY	GPY	PFP_N_	^G^RE_N_	RE_N_
STSNT	1								
LNT	0.923**	1							
STSNE	0.407*	0.641**	1						
LNE	0.729**	0.625**	0.031	1					
GY	0.650**	0.400	−0.351	0.750**	1				
GPY	0.871**	0.676**	−0.019	0.775**	0.915**	1			
PFP_N_	0.334	−0.278	−0.068	0.775**	0.432	0.453	1		
^G^RE_N_	0.569*	−0.313	−0.195	0.642**	0.547*	0.580*	0.877**	1	
RE_N_	0.582*	−0.186	−0.110	0.590**	0.505*	0.552*	0.885**	0.951**	1

## Discussion

### N accumulation and distribution in response to N application

High levels of N, ranging from 600 to 800 kg N ha^−1^, have been applied annually for intensive agriculture over several decades in the NCP, along with excessive irrigation (300–400 mm) during the wheat growing season in farmers’ fields, leading to a rapid decline in the groundwater table and challenges in sustainability^24–26^. Therefore, the optimization of N supply to wheat requirements should consider moderate irrigation methods on the basis of water-saving in this area. N accumulation and redistribution are crucial when determining grain quality and yield^27,28^. In leaves, amino acids in proteins are broken down into free amino acids that are stored in leaves and then transported to the grains through the stem, influencing the grain protein content^29^. In this study, treatment with 180 kg N ha^−1^ maintained a higher level of free amino acid content in the flag leaf and grain after anthesis compared with 0 kg N ha^−1^ (Fig. 1A,B). This resulted in higher N being distributed in grain at maturity leading to the increase in grain N protein concentration, which indicated that N input accelerated the export of more free amino acids to grains, where it was beneficial for protein synthesis. Approximately 60–95% of the grain N at maturity relies on the remobilization of N stored in the shoots and roots of wheat before anthesis^11,30^, which is influenced by water condition, N supply, and genotype^31,32^. Following application of N at 200 kg ha^−1^, the vegetative organs of wheat were found to transfer 84.0% of stored N to the ears during grain filling, which reduced to 79.0% at 0 kg N ha^−1^ ^33^. N remobilization in durum wheat plants were increased by N availability and decreased by water stress^34^. When N was supplied at 240 kg N ha^−1^, SI based on soil moisture in the 0–40 cm soil layer improved N distribution from vegetable organs to grains^35^. In our study, soil water content with 70% and 65% of FC at jointing and anthesis were achieved under SI by measuring the moisture in the 0–40 cm soil layer, 180 kg N ha^−1^ obtained a higher N translocation efficiency in the STS and leaf than other N treatments (Table 2), and did not decrease the N distributed in the spike stalk and shell and grains at maturity compared with 240 kg N ha^−1^ and 300 kg N ha^−1^ (Fig. 2). This suggests that enhancing the translocation efficiency of N fertilizer at a low level of N application is a good method to improve the grain N accumulation of wheat.

### Responses of soil NO3-N residue to N application

High residual NO_3_-N in the 0–200 cm soil profile in the majority of farmland in China is the result of a long period of heavy N application^36^. Furthermore, excessive fertilization can contribute to a 6–19% reduction in apparent N recovery efficiency, and a 30–93% increase in soil NO_3_-N residue^37^. Irrigation also affects nitrate leaching^38^, and nitrate leaching into groundwater has been found to account for 29.7–47.9% of applied N following irrigation from 104 to 400 mm and the application of N fertilization from 104 to 400 kg ha^−1^ ^39^. SI with suitable SRWC in the 0–40 cm soil layer can reduce NO_3_-N leaching, resulting in a higher output of soil N^35^. In the present study, the NO_3_-N residue in the 0–180 cm soil profile increased with increasing N application in both years (Fig. 3). Application of 180 kg N ha^−1^ reduced the soil NO_3_-N residue in the 60–180 cm layer compared with application of 240 and 300 kg N ha^−1^ under SI in both years (Fig. 3). This indicates that leaching of NO_3_-N at deeper soil depths can be reduced with application of 180 kg N ha^−1^, which might be due to the lower N supply as well as the higher N translocation efficiency in the STS and leaf in winter wheat during the growing stages.

### Responses of yield and N utilization to N application

Crop yield is strongly influenced by N fertilizer and varies due to differences in the soil water content^40,41^. N input at 100 kg N ha^−1^ with 120 mm increased the spike number and 1000-grain weight, leading to a higher grain yield compared with N input at 200 kg N ha^−1^ with 180 mm^15^. At 80 or 70% full irrigation, a significant increase in yield was recorded up to 80 kg N ha^−1^, while under water-limiting conditions (60 or 50% full irrigation), a significant increase in yield was only recorded up to 40 kg N ha^−1^ ^42^. SI can clearly affect the soil moisture content^43^. In our study, under SI based on RSWC, reducing the N input to 180 kg N ha^−1^ maintained the grain yield at a high level for 2 years (7.67 t ha^−1^ in 2015/2016, 8.83 t ha^−1^ in 2016/2017) (Table 3). This was mainly attributed to the increase in spike number and the grain number per spike; there was no significant increase in grain yield when N input increased to 240 and 300 kg ha^−1^. Similar 1000-grain weights were observed with 180 and 0 kg N ha^−1^ in 2015/2016, but were lower than that of 0 kg N ha^−1^ in 2016/2017 (Table 3). This may be due to the marked increase in spike number per m^2^ in 180 kg N ha^−1^, and monthly precipitation fluctuations (Table 3; Fig. S1). As yield components are usually reported to provide a snapshot of the final yield composition^44^, the number of spikes per m^2^ is somewhat driven by environmental factors^45^.

Higher grain N content and lower loss of N lead to higher N use efficiency, contributing to a more optimal N balance^3^. In our study, 180 kg N ha^−1^ did not significantly decrease the grain yield or grain protein concentration, and there was no decrease in grain protein yield compared with 240 and 300 kg N ha^−1^. Furthermore, 180 kg N ha^−1^ significantly increased the PFP_N_ compared with 240 and 300 kg N ha^−1^ and obtained the highest ^G^RE_N_ and RE_N_. Wheat were able to tolerate 180 kg N ha^−1^, with no decrease in grain yield or grain protein yield, since ‘non-detrimental’ deficiencies can enhance the environmental and economic performance of wheat by increasing N use efficiency^46^. Additionally, leaf N translocation efficiency was positively correlated with grain yield, grain protein yield, PFP_N_, ^G^RE_N_, and RE_N_ (Table 4), indicating that improving N translocation efficiency in the leaf may be conducive to the increase grain yield, grain protein yield, and N utilization. Grain yield was positively correlated with RE_N_, similar to the results reported by Ye *et al*.^47^. N translocation efficiency in the STS was not closely related to grain yield or grain protein yield (Table 4). This may be because most reserves are stored in the stem and only remobilized with low efficiency, and dry matter of stem requires a minimum protein content to retain the function of material transportation and provide mechanical support^14^. We conclude that under SI, 180 kg N ha^−1^ is an appropriate N fertilization rate to maintain high grain yield, grain protein yield and high N use of winter wheat in the NCP.

## Conclusion

Under SI with 70% RSWC at jointing and 65% RSWC at anthesis, 180 kg N ha^−1^ promoted an increase in grain yield, grain protein yield, and N use in winter wheat by increasing the free amino acid content in the flag leaf and grain after anthesis; the dry matter and plant N accumulation, and N translocation in the STS and leaf, which also led to higher N distribution in grains. N translocation efficiency in leaf was positively related to grain yield, grain protein yield, PFP_N_, ^G^RE_N_, and RE_N_. Application of N at 180 kg N ha^−1^ was optimal for wheat production, and could maintain the grain yield over 7.5 t ha^−1^ for 2 years. Additional N input did not further increase the grain yield and grain protein yield, but decreased the PFP_N_, ^G^RE_N_, RE_N_ and increased the soil NO_3_-N residue of the 60–180 cm soil layer in both seasons, which had potentially unfavorable effects on the environment.

## Material and Methods

### Experimental site

Field experiments were carried out between 2015 and 2017 at the experimental farm of Shandong Agricultural University (36°09′N, 117°09′E), Tai’an, China. This study area has a temperate, semi-humid, and continental monsoon climate, with an annual average temperature of 12.9–13.6 °C and average annual precipitation of 500–800 mm. Before winter wheat was sown, organic matter, total N, hydrolysable N, available phosphate, and available potassium (K) in the surface soil (0–20 cm) were 1.50%, 0.15%, 117.69 mg kg^−1^, 41.58 mg kg^−1^, and 133.86 mg kg^−1^, respectively. The total amounts of precipitation during the wheat growing season in 2015/2016 and 2016/2017 were 184.2 and 180.8 mm, respectively, and monthly total precipitation and mean temperature are shown in Supplementary Fig. S1.

### Experimental design

N was applied at four rates: 300 (N_300_, traditional rate of N applied by farmers); 240 (N_240_, 80% of the N application rate N_300_); 180 (N_180_, 60% of the N application rate N_300_); and 0 (N_0_) kg N ha^−1^. N fertilizer was applied as urea (46% N content) twice: 50% of N fertilizer was applied as basic N fertilizer to the experimental plots before sowing, and the remaining N was ditched at jointing. The SI for each N treatment was 70 and 65% relative soil moisture contents at the jointing and anthesis stages, respectively, in the 0–40 cm soil layer. The amount of irrigation was calculated using the equation^48^: IM = 10 × ρb × D × (θi-θj), where IM (mm) is the amount of SI; ρb (g cm^−3^) is the soil bulk density; D (cm) is the soil profile depth measured for SWC before irrigation (40 cm); θi (%) is the target SWC on a weight basis after SI (field capacity × targeted relative soil water content); and θj (%) is the SWC on a weight basis before SI. A rotor flowmeter (Huanxiang DN40, China) was used to measure the amount of applied water.

All treatments were applied in triplicate in a randomized design. Each experimental plot was 2 × 10 m in size with a 1.0 m buffer zone between plots to minimize the effects of adjacent plots.

### Crop management

Jimai 22, one of the most widely planted wheat cultivars in the NCP, was used in this study. Base fertilizer of 150 kg ha^−1^ P_2_O_5_ and 112.5 kg ha^−1^ K_2_O was applied before sowing. Wheat seeds were sown at a density of 225 plants m^−2^ on 13 October 2015 and 11 October 2016, and were harvested on 10 June 2016 and 9 June 2017.

### Sampling and analysis

#### Plant sampling and analysis

Twenty flag leaves and spikes were sampled 0, 7, 14, 21, and 28 days after anthesis. Fresh samples were immediately frozen in liquid nitrogen and stored at -40 °C prior to use in free acetic acid assays. For this, 0.5 g of fresh samples (flag leaves and grains) was ground with 5 mL of 10% acetic acid and then centrifuged at 10,000 × g and 4 °C for 10 min. Then, 0.5 mL of supernatant was made up to a volume of 25 mL with pH 5.4 acetate buffer to produce the extract, and was then heated in boiling water for 15 min. The free amino acid content was assayed by the ninhydrin method^49^.

Twenty consecutive plants were collected in each plot to estimate dry matter accumulation at pre-winter, revival, jointing, anthesis and maturity of wheat growing stages. At anthesis, the samples were separated into leaves, stems, and glumes, whilst those at maturity were separated into leaves, stems, glumes, and grains. All samples were oven-dried to a constant weight at 80 °C, after heating at 105 °C for 30 min, and then weighed to determine dry matter.

Next, all samples were milled into powder, which was subsequently used to determine the N concentration using the Kjeldahl method. The grain protein content was calculated by multiplying the grain N concentration by the conversion factor, 5.7. The parameters related to N accumulation and remobilization within the winter wheat were calculated as described by Papakosta and Gagianas^31^ and Wang *et al*.^50^ as follows:

N accumulation of an organ (kg ha^−1^) = N concentration of the organ × dry weight of the organ;

N accumulation in a plant = total N accumulation of all organs at a certain growth stage;

N translocation amount of an organ (kg ha^−1^) = N accumulation of the organ at anthesis − N accumulation of the organ at maturity;

Nitrogen translocation efficiency of an organ (%) = (N translocation amount of the organ/N accumulation of the organ at anthesis) × 100;

Contribution of N translocation amount from the vegetative organ to the grain (%) = (N translocation amount in vegetative organ/grain N accumulation at maturity) × 100.

Grain yield was determined by all plants harvested in a 2 m^2^ area of each plot and recorded at a 12.5% moisture content. All spikes from the sampled area were counted to estimate spike number ha^−1^ for each plot. Twenty stems from each plot were randomly selected to determine the grain number per spike. The 1000-grain weight was determined using grains harvested from each sampled area.

The grain protein yield, N partial factor productivity (PFP_N_), grain N recovery efficiency (^G^RE_N_), and N recovery efficiency (RE_N_) were calculated as follows:

Grain protein yield (t ha^−1^) = grain protein content × grain yield;

PFP_N_ (kg kg^−1^) = grain yield in treatments with N application/amount of applied N;

^G^RE_N_ (%) = (grain N accumulation in treatments with N application − grain N accumulation in N_0_)/amount of N applied × 100;

RE_N_ (%) = (total N accumulation in plant in treatments with N application − total N accumulation in plant in N_0_)/amount of N applied × 100.

#### Soil sampling and analysis

Soil samples were collected from each 20 cm thick soil layer, vertically down to 200 cm using a soil corer at five points in each plot at maturity. Samples collected from the same soil layer in the same plot were mixed, placed into polyethylene bags, sealed, and then frozen at −20 °C before laboratory extraction. Three representative subsamples from each soil layer were extracted using 0.01 mol L^−1^ CaCl_2_ solution (NATESC, 2006). NO_3_-N concentration were measured using an ultraviolet spectrophotometer (TU-1901, Presee, China). Soil NO_3_-N (kg ha^−1^) residue was calculated by the following Equation^37^:

$${{\rm{C}}}_{{\rm{NO}}3}={\rm{H}}\times {\rm{\rho }}{\rm{b}}\times {\rm{c}}/10$$

where, C_NO3_ (kg ha^−1^) is NO_3_-N residue, H (cm) is soil thickness; ρb (g cm^−3^) is the soil bulk density measured in undisturbed soil samples from each soil layer using 100 cm^3^ rings; and c (mg kg^−1^) is the NO_3_-N concentration.

### Statistical analysis

Statistical analyses were performed using standard analysis of variance (ANOVA) in SPSS 22.0. The normality of data and the homogeneity of variances were checked by the Levene and Shapiro-Wilk tests, respectively. An ANOVA was performed to compare the effects of different treatments on the measured variables. To identify significant effects, the means were compared by Duncan’s test at α = 0.05. Two-way ANOVA was performed where N treatments and year were used as main factors. A linear-plateau model was fitted with SPSS 22.0 to obtain the response of grain yield to N application rate. Two-tailed Pearson correlation analyses were performed to reveal the relationships among N translocation, grain yield, grain protein yield, PFP_N_, ^G^RE_N_ and RE_N_ in a combined analysis of data over 2 years.

## Supplementary information

Supplementary Information.

## Data Availability

All data generated or analyzed during this study are included in this published article (and its Supplementary Information files).
