# Supplementary material for: Nitrogen supply modulates nitrogen remobilization and nitrogen use of wheat under supplemental irrigation in the North China Plain
Source: Sci Rep. 2020 Feb 24;10:3305. doi: 10.1038/s41598-020-59877-5 (PMC7039916; doi:10.1038/s41598-020-59877-5)
Supplement: Supplementary file 1 — Supplementary Information. [file 41598_2020_59877_MOESM1_ESM.pdf]

Supplementary Material for

Nitrogen supply modulates nitrogen remobilization and nitrogen use of wheat under supplemental irrigation in the North China Plain

Xuejiao Zheng, Zhenwen Yu, Yongli Zhang*, Yu Shi

This file includes:
Figure S1.

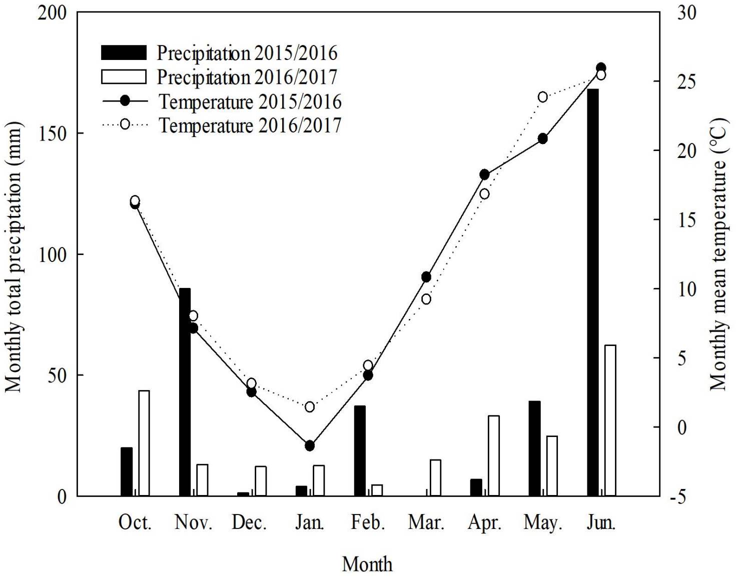

Figure S1. Monthly total precipitation and mean temperature recorded during the wheat growing season in 2015/2016 and 2016/2017.
